# Dementia Risk and Gender Equality: Global Insights Into Social Determinants

**DOI:** 10.1111/nhs.70230

**Published:** 2025-09-16

**Authors:** Wenpeng You

**Affiliations:** ^1^ School of Biomedicine The University of Adelaide Adelaide Australia; ^2^ School of Nursing and Midwifery Western Sydney University Sydney Australia

**Keywords:** dementia gender disparity, dementia incidence, gender equality, lifestyle shifts, women's health risks

## Abstract

This retrospective cross‐sectional study analyzed data from 2011 to 2021 across 154 countries to explore the relationship between gender equality, measured by the Gender Equality Index (GEI), female dementia incidence, and gender disparities at global and regional levels. Higher GEI scores were linked to elevated dementia incidence among women and an expanding female–male disparity, with the strongest associations observed in resource‐limited countries undergoing rapid social and lifestyle transitions. Multiple regression identified GEI as the most significant predictor of both female dementia incidence and gender disparity, while economic affluence and urbanization showed no independent effects. Findings highlight a paradox: although gender equality advances women's access to education, employment, and healthcare, it may simultaneously increase exposure to dementia‐related risk factors, including chronic stress, work–life imbalance, and lifestyle changes such as greater smoking and alcohol use. These results emphasize the importance of gender‐sensitive public health strategies that address unintended health consequences of social progress. Interventions should prioritize stress management, workplace mental health, and lifestyle modification while accounting for disproportionate caregiving responsibilities, thereby supporting equitable cognitive health outcomes for women worldwide.

**Summary**

- Higher Gender Equality Index (GEI) scores are significantly associated with increased female dementia incidence and widening gender disparities.
- The strongest associations are observed in low‐ and middle‐income countries undergoing rapid social and lifestyle transitions.
- GEI emerged as the most significant predictor of dementia risk and disparity, while urbanization and national income showed no independent effects.
- Rising gender equality may inadvertently increase women's exposure to dementia‐related risk factors such as chronic stress, work–life imbalance, smoking, and alcohol consumption.
- Gender‐sensitive public health strategies are needed to address the paradoxical health consequences of social progress, emphasizing stress management, workplace mental health, and caregiving support for women.

**Abbreviations**
DIR: dementia incidence rate
GDP PPP: gross domestic product per capita purchasing power parity
GEI: gender equality index
GII: gender inequality index
I_bs_
: biological state index, measuring dementia genetic background accumulation at population level
IHME: Institute for Health Metrics and Evaluation of the University of Washington
UN: United Nations
WHO: World Health Organization

## 1 Introduction

Dementia is a major global health issue which significantly affects more women than men (IHME 2020; WHO 2023a). This statistically significant gender disparity can be explained by a combination of biological, social, and lifestyle factors. Biologically, women tend to live longer than men, which increases their cumulative risk of developing dementia (Bailey‐Taylor et al. 2022; Shaw et al. 2021; WHO 2023b). Hormonal differences may also contribute to sex‐specific vulnerabilities (Bassani et al. 2023; Russell et al. 2019; Zhu et al. 2021). Socially, women are more likely to assume caregiving roles (Pusswald et al. 2015; Vitaliano et al. 2011), experience greater exposure to stress (Guo et al. 2024; Hasselgren et al. 2020), and historically have had lower access to education (Alty et al. 2023), all of which are associated with an increased risk of dementia (Geraets and Leist 2023; IHME 2020). The number of women affected by dementia is expected to grow due to the aging global population, highlighting the urgent need to understand gender‐specific risk factors for dementia (Gong et al. 2023; Guo et al. 2024).

Risk factors linked to dementia such as age, genetics, lifestyle habits, and socio‐economic conditions have been studied extensively (Ou et al. 2024). More recently, there has been growing interest in understanding how structural determinants, such as gender equality, may indirectly influence dementia risk among women (Avila‐Rieger et al. 2025; Moutinho 2025).

As societies become more gender‐equal, women gain greater access to education, employment, and financial independence, enabling broader lifestyle choices and greater autonomy (Kumar et al. 2024). While these developments are empowering, they may also coincide with increased exposure to behaviors traditionally associated with men, such as alcohol consumption, smoking, sedentary lifestyles, and participation in high‐stress occupations (Cattani and Rizza 2024; Roczen et al. 2024; Verplaetse et al. 2025). Although such behaviors are more prevalent in lower socioeconomic groups, emerging evidence suggests that rising gender equality in middle‐ and high‐income settings can contribute to their uptake among women across various socioeconomic backgrounds (Bilal et al. 2016; Graham 2009). These behaviors are associated with increased risks of cardiovascular disease, diabetes, and other chronic conditions that are well‐established contributors to dementia risk (Lu et al. 2021; Shang et al. 2022; Wang et al. 2024).

The Gender Equality Index (GEI), derived from the Gender Inequality Index (GII), is a comprehensive, multidimensional measure of gender parity within a country and encompasses three core domains: reproductive health (maternal mortality ratio and adolescent birth rates), empowerment (proportion of women with at least secondary education and representation in parliament), and labor market participation (Berik 2022; UNDP 2024). These indicators collectively reflect the structural, social, and economic positioning of women in society (Mejia‐Arango et al. 2021). Their relevance to dementia lies in their close association with health behaviors, chronic stress exposure, and access to resources throughout the life course (Guo et al. 2024). For example, while higher educational attainment and workforce participation can promote autonomy and healthcare access, they may also increase exposure to occupational stressors and lifestyle risks. In addition, delayed childbirth and smaller family sizes, more common in gender‐equal societies, may influence hormonal exposure, particularly estrogen, which plays a role in cognitive health (Zhang et al. 2023).

By incorporating the GEI as a composite indicator, this study captures the broader societal context in which women live, work, and age, offering a comprehensive lens to explore how gender equality may inadvertently contribute to female dementia risk (Ribeiro et al. 2023). Despite these plausible pathways, limited research has systematically examined how structural gender equality, as reflected in GEI dimensions, may influence dementia incidence among women through changing roles and lifestyle factors (Gilles et al. 2024). Understanding this relationship is critical for informing gender‐sensitive public health strategies, particularly in countries undergoing rapid social and economic transformation (Petersen et al. 2021).

The connection between psychological well‐being and physical health is well established, with research showing that negative emotions such as chronic stress, anxiety, and depression can contribute to biological changes, such as elevated cortisol levels and inflammation that impair immune function and increase vulnerability to chronic conditions, including dementia (Du et al. 2024). For example, research has consistently shown that negative life events, such as the death of a loved one, divorce, or personal injury, along with high‐stress lifestyles, depression, and anxiety can contribute to dementia risk (Franks et al. 2021; Severs et al. 2023). These psychological stressors are particularly relevant for women, who may experience unique forms of stress due to societal expectations and caregiving responsibilities (Viertiö et al. 2021). The cumulative effects of these stressors over a lifetime can increase the risk of dementia, highlighting the need to consider psychological well‐being alongside biological and social factors (Wallensten et al. 2023).

Historically, dementia epidemiology has primarily emphasized biological risk factors such as age, genetics, and neuropathological changes. However, increasing attention is now being paid to social, psychological, and environmental determinants that also influence dementia risk (Brenowitz et al. 2021; Da et al. 2024). Gender equality and evolving gender roles may reinforce disparities in dementia risk between men and women (Mielke et al. 2022). Greater gender equality can empower women and improve access to healthcare and economic resources, which are protective for overall health. Yet, it may also introduce new risks through changing lifestyles and stress exposures (Chelberg and Steele 2024; Hasselgren et al. 2020; Park et al. 2013; WHO 2021).

This study explores the relationship between gender equality, as measured by the GEI (UNDP 2024a), and female dementia incidence rates globally. Specifically, I investigated whether higher GEI scores were associated with increased dementia incidence among women and greater gender disparities. I also examined whether these associations differ by economic development and region, with particular attention to low‐ and middle‐income countries where rapid social transformations may have profound implications for women's health. By addressing this gap, the study highlights the paradoxical relationship between social progress and health risks, providing evidence to inform gender‐sensitive dementia prevention strategies worldwide.

## 2 Materials and Methods

This retrospective cross‐sectional study analyzed population‐level data across 154 countries to examine the association between gender equality and dementia incidence among women.

### 2.1 Data Sources

Data were compiled from internationally recognized repositories: the Institute for Health Metrics and Evaluation (IHME) for sex‐specific dementia incidence rates (IHME 2020); the United Nations Development Programme (UNDP) for the Gender Inequality Index (GII) (UNDP 2024); the World Bank for economic and demographic indicators (World Bank 2024; You et al. 2022); and previously published literature for the Biological State Index (I_bs_), which measures the accumulation of deleterious alleles due to reduced natural selection (You and Henneberg 2022). These sources provide comprehensive, validated, and comparable cross‐country data.

### 2.2 Study Variables

#### 2.2.1 Data Sources and Study Design

To ensure comprehensive coverage, data were obtained from publicly accessible repositories, including previous peer‐reviewed publications, the World Bank, and the IHME (IHME 2020). These sources provide reliable and harmonized datasets for demographic and health analyses at the global level.

#### 2.2.2 Dependent Variables

The female dementia incidence rate (FDIR) was selected as the primary dependent variable, as it captures new dementia cases and offers a more accurate measure of risk than prevalence rates. A secondary dependent variable, dementia gender disparity, was constructed by subtracting male dementia incidence from female dementia incidence. This measure allowed the author to evaluate how gender equality predicts the extent to which women experience disproportionately higher dementia incidence compared to men.

#### 2.2.3 Independent and Confounding Variables

The primary independent variable was the Gender Equality Index (GEI) for 2011, calculated from the United Nations Development Programme's Gender Inequality Index (GII) using the formula: GEI = 1‐GII.

A higher GEI value represents greater gender equality (UNDP 2024). The GEI reflects inequalities in education, political representation, and labor market participation. Such structural inequalities can increase chronic stress among women, which is known to negatively affect mental health and contribute to the progression of chronic diseases. Recent evidence also suggests that stress may be a contributing factor to dementia risk (Yu et al. 2023).

Four confounders were included:

- **Genetic predisposition**, measured by the Biological State Index (I_bs_) (You and Henneberg 2022; You et al. 2022);
- **Economic affluence**, represented by GDP per capita (World Bank 2018, 2024);
- **Urbanization**, measured as the percentage of the population living in urban areas (2018) (World Bank 2022);
- **Female aging**, proxied by life expectancy at birth in 2018 (Speechly et al. 2008; WHO 2023a; You and Henneberg 2022).

### 2.3 Temporal Design and Rationale

Dementia develops over the life course, influenced by cumulative exposures, so we employed a time‐lagged design to capture temporal dynamics. GEI data from 2011 were selected to reflect earlier structural and societal conditions, acknowledging that shifts in gender roles, education, employment, and lifestyle behaviors may take years to influence dementia risk. Dementia incidence data from 2021 were then aligned with these exposures to account for the delayed manifestation of outcomes. Confounder data from 2018 were incorporated as they represent the most reliable, globally harmonized estimates available prior to 2021 and generally change gradually over time. This staggered temporal alignment reduces the risk of reverse causality, avoids reliance on short‐term fluctuations, and supports the identification of long‐term patterns, thereby strengthening the ecological validity of the analysis (Kim et al. 2021; Poppe et al. 2025).

### 2.4 Data Selection and Sample

Since this study relies on population‐level data, individual patient recruitment and traditional flow diagrams are not applicable. Instead, a data selection flowchart (Data S1) was developed to illustrate the inclusion and exclusion process. Initially, 204 populations with dementia incidence data (female and male) from IHME were screened. Countries were retained if both dementia incidence data and corresponding GEI values were available. A case‐wise deletion method excluded countries with > 5% missing data in key variables to ensure analytical consistency. This process yielded a final analytic sample of 154 countries, representing both high‐income and low‐ and middle‐income countries (LMICs), as defined by World Bank classifications.

### 2.5 Terminology

The reporting units vary across data sources. IHME uses “location,” the World Bank uses “economy,” and the genetic index source uses “country.” While not all units denote sovereign states, for clarity we use the terms “population” and “country” interchangeably in this study.

### 2.6 Data Analysis

Regression diagnostics assessed multicollinearity prior to correlation and regression analyses. Tolerance and variance inflation factor (VIF) values for six key variables (GEI, FDIR, female aging, economic affluence, genetic predisposition, urbanization) met accepted thresholds (tolerance ≥ 0.10, VIF ≤ 10), indicating no concerns. Repeating the analysis with dementia gender disparity as the dependent variable produced similarly acceptable results (O'brien 2007).

With the same set of raw data, correlation and regression analyses proceeded in five steps using six approaches (You and Henneberg 2022; You et al. 2022):

Scatter plots were produced in Excel (Microsoft 2016) to explore and visualize the correlations between GEI and FDIR. Scatter plots also allowed us to assess data quality and distributions of the variables.

Bivariate Correlation (Pearson's r and non‐parametric) analyses were conducted to examine the strength and direction of the correlations between all the six variables. This allowed the author to examine the correlated variables from common sense and to align the correlations identified in previous studies to ensure the data quality. For example, it also allowed the author to check if the potential confounding variables were chosen properly.

Partial correlation: I alternated each of the five variables (GEI, aging, economic affluence, genetic predisposition and urbanization) as the independent predictor when all the other four variables were included as the potential confounding factors. And then, I alternated each individual variable as the control variable to assess the relationships between FDIR and other four variables respectively.

Multiple linear regression: Standard and stepwise regression visualized the correlation between DIR and each predictor, identifying the most significant predictors.

Grouped country nonparametric correlation analysis: To understand the GEI‐ FDIR correlation across diverse contexts, countries were grouped by various criteria: World Bank income classifications, UN common practice and WHO regions, and other socio‐economic or geographic groupings (e.g., ACD, APEC, EEA, OECD). These groupings, matched with DIR data, allowed for a nuanced exploration of how different factors influence dementia incidence globally.

The above dependent variable, FDIR was replaced with gender disparity for repeating and exploring if higher‐GEI countries had significantly greater female incidence rate than male incidence rate.

Data analysis was performed using SPSS v. 29. Significance was reported for *p* < 0.05, < 0.01, and < 0.001. Regression criteria were set with a probability of F to enter ≤ 0.05 and to remove ≥ 0.10. Scatter plots were created using raw data in the Microsoft Excel 2016.

## 3 Results

**FIGURE 1:**
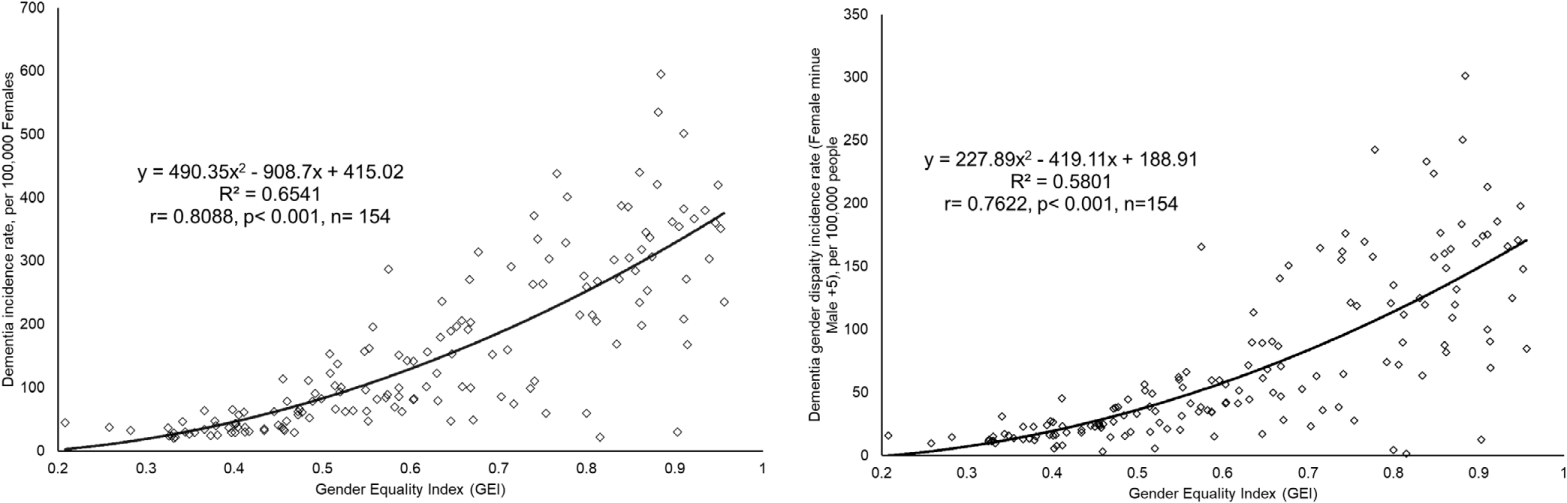
The relationship between the Gender Equality Index and dementia incidence, including both female‐specific rates and the difference in incidence between females and males. (a) The relationship between the Gender Equality Index and the female dementia incidence rate. (b) The relationship between the Gender Equality Index and the dementia incidence difference (Female–Male). Data source and definition: The Gender Equality Index (GEI) is derived using the formula: GEI = 1‐Gender Inequality Index, with the Gender Inequality Index sourced from the United Nations Development Programme (2024); Dementia Incidence Rates are sex‐specific and reported as the number of new cases per 100 000 individuals diagnosed in 2021, sourced from the Institute for Health Metrics and Evaluation, University of Washington. Dementia gender disparity refers to the difference in dementia incidence rates between females and males, calculated as the female incidence rate minus the male incidence rate.

Figure 1 illustrates the relationship between the GEI and female dementia incidence, focusing on female‐specific rates and gender disparities. In Figure 1a, as GEI increases, indicating higher gender equality, shifts in female dementia incidence rates were evident, suggesting that rising gender equality may influence women's dementia risk through lifestyle, autonomy, and role changes. Overall, GEI accounts for 65.41% of the variance in FDIR. Figure 1b examines the difference in dementia incidence between females and males (female rate minus male rate) across GEI levels, highlighting how increased gender equality may affect gender disparities in dementia incidence, with GEI explaining 58.01% of the variance in dementia gender disparity.

Table 1 shows that the GEI shows strong positive correlations with Female Dementia Incidence (FDIR) (*r* = 0.799) and Dementia Gender Disparity (*r* = 0.752), indicating that GEI is linked to increased dementia rates among women and a wider gap between female and male cases. GEI also correlates with Genetic Predisposition (*r* = 0.719) and Female Aging (*r* = 0.785), suggesting that countries with GEI tend to have populations with greater genetic susceptibility and longer female lifespans. Additionally, FDIR is nearly perfectly correlated with dementia gender disparity (*r* = 0.962) and is positively related to economic affluence (*r* = 0.581), genetic predisposition (*r* = 0.590), and Female Aging (*r* = 0.724). A notably high correlation between Genetic Predisposition and Female Aging (*r* = 0.893) further emphasizes their combined impact, while urbanization shows a more indirect influence.

**TABLE 1:** Combined correlation matrix and descriptive statistics for the seven variables.

Variable	Correlation matrix for the seven variable							Descriptive statistics for all the seven variables				
	Gender equality index	Female dementia incidence	Dementia gender disparity (female minus male)	Aging, female	Economic affluence	Genetic predisposition	Urbanization	*N*	Minimum	Maximum	Mean	Std. deviation
Gender equality index	1.000	0.799***	0.752***	0.785***	0.707***	0.719***	0.520***	154	0.21	0.96	0.62	0.19
Female dementia incidence	0.834***	1.000	0.970***	0.720***	0.558***	0.602***	0.485***	154	20.44	595.69	158.39	131.31
Dementia gender disparity (female minus male)	0.801***	0.948***	1.000	0.634***	0.496***	0.524***	0.418***	154	−3.43	296.42	65.86	63.99
Aging, female	0.840***	0.812***	0.729***	1.000	0.719***	0.895***	0.654***	153	52.77	87.32	75.55	7.66
Economic affluence	0.825***	0.737***	0.671***	0.903***	1.000	0.586***	0.665***	148	758	116 499	22 781	21 984
Genetic predisposition	0.886***	0.828***	0.746***	0.942***	0.908***	1.000	0.599***	152	0.63	0.99	0.93	0.08
Urbanization	0.533***	0.534***	0.466***	0.688***	0.759***	0.679***	1.000	154	13.03	100.00	60.95	22.39

*Note:* Nonparametric (below diagonal) and Pearson r (above diagonal) correlations were reported. Data source & definition: Gender Inequality Index (GII), United Nations Development Programme (2011); Dementia incidence rate (DIR), the number of new cases per 100 000 people diagnosed in 2019 Institute for Health Metrics and Evaluation of University of Washington; Economic affluence, per capita GDP PPP, measured with the per capita purchasing power parity (PPP) value of all final goods and services produced within a territory in a given year (2018), the World Bank; Biological State Index (I_bs_), dementia genetic background accumulation level due to reduced natural selection, downloaded from previous publication (You and Henneberg 2022); Life expectancy at birth, Female (Life e(0)), the World Bank 2018; Urbanization, measured with the percentage of population living in urban area in 2018, the World Bank. Significance levels: ***p < 0.001. Number of country range, 148–154.

Table 1 also represents that across 154 countries, GEI values range from 0.21 to 0.96 (mean = 0.62, SD = 0.19), reflecting moderate‐to‐high gender equality. Female Dementia Incidence ranges widely from 20.44 to 595.69 new cases per 100 000 (mean = 158.39, SD = 131.31), and dementia gender disparity averages 65.86 (range: −3.43 to 296.42, SD = 63.99), indicating that, on average, female incidence exceeds male incidence by about 66 cases per 100 000. Female life expectancy ranges from 52.77 to 87.32 years (mean = 75.55, SD = 7.66). Economic affluence shows substantial variability (range: 758 to 116 499; mean = 22 781, SD = 21 984), genetic predisposition is consistently high (range: 0.63 to 0.99; mean = 0.93, SD = 0.08), and Urbanization averages 60.95% (range: 13.03% to 100.00%, SD = 22.39%).

Table 2a displays the partial correlation coefficients for female dementia incidence (FDIR) when each predictor is alternated while holding the others constant. Here, the GEI exhibits a strong positive correlation with FDIR (*r* = 0.585, *p* < 0.001), suggesting that GEI is independently associated with increased dementia incidence among women. Female aging also shows a modest yet significant positive correlation (*r* = 0.185, *p* < 0.05), indicating that longer female life expectancy contributes to higher dementia rates. In contrast, genetic predisposition (*r* = −0.141, *p* = 0.092), economic affluence (*r* = −0.131, *p* = 0.119), and urbanization (*r* = 0.046, *p* = 0.586) exhibit weak or non‐significant relationships with female dementia incidence.

**TABLE 2:** Comparative partial correlation coefficients for female dementia incidence rate and predicting variables across various combinations of controlled variables.

Variables	(a) Gender equality index, genetic predisposition, economic affluence, urbanization and Life e (0) were alternated as the predicting variable for calculating its independent relationship with dementia incidence while the other four variables are statistically kept constant														
	Dementia incidence, female			Dementia incidence, female			Dementia incidence, female			Dementia incidence, female			Dementia incidence, female		
	*r*	*p*	df	*r*	*p*	df	*r*	*p*	df	*r*	*p*	df	*r*	*p*	df
Gender equality index	0.585	< 0.001	142	—	—	—	—	—	—	—	—	—	—	—	—
Genetic predisposition	—	—	—	−0.141	0.092	142	—	—	—	—	—	—	—	—	—
Economic affluence	—	—	—	—	—	—	−0.131	0.119	142	—	—	—	—	—	—
Urbanization	—	—	—	—	—	—	—	—	—	0.046	0.586	142	—	—	—
Aging, female, 2018	—	—	—	—	—	—	—	—	—	—	—	—	0.185	< 0.050	142

Variables	(b) Gender equality index, genetic predisposition, economic affluence, urbanization and life e (0) were alternated as the predicting variable for calculating its independent relationship with dementia incidence difference (female—male) while the other four variables are statistically kept constant														
	Dementia incidence difference (female–male)			Dementia incidence difference (female—male)			Dementia incidence difference (female—male)			Dementia incidence difference (female—male)			Dementia incidence difference (female–male)		
	*r*	*p*	df	*r*	*p*	df	*r*	*p*	df	*r*	*p*	df	*r*	*p*	df
Gender equality index	0.568	< 0.001	142	—	—	—	—	—	—	—	—	—	—	—	—
Genetic predisposition	—	—	—	−0.111	0.187	142	—	—	—	—	—	—	—	—	—
Economic affluence	—	—	—	—	—	—	−0.123	0.141	142	—	—	—	—	—	—
Urbanization	—	—	—	—	—	—	—	—	—	0.026	0.755	142	—	—	—
Female aging, 2018	—	—	—	—	—	—	—	—	—	—	—	—	0.091	0.277	142

*Note:* Data source & definition: Gender Inequality Index (GII), United Nations Development Programme, 2011; [Dementia incidence difference (Female – Male) (the difference in dementia incidence rates between females and males), with sex‐specific dementia incidence rates reported as the number of new cases per 100 000 individuals diagnosed in 2019, sourced from the Institute for Health Metrics and Evaluation, University of Washington] Dementia incidence rate (DIR), the number of new cases per 100 000 people diagnosed in 2019 Institute for Health Metrics and Evaluation of University of Washington; Economic affluence, per capita GDP PPP, measured with the per capita purchasing power parity (PPP) value of all final goods and services produced within a territory in a given year (2018), the World Bank; Biological State Index (I_bs_), dementia genetic background accumulation level due to reduced natural selection, downloaded from previous publication (You and Henneberg 2022); Life expectancy at birth, Female (Life e(0)), the World Bank 2018; urbanization, measured with the percentage of population living in urban area in 2018, the World Bank.

Table 2b reports the partial correlation coefficients for the dementia incidence difference between females and males. In this analysis, GEI remains a strong predictor (*r* = 0.568, *p* < 0.001), implying that GEI is linked to a larger gap in dementia incidence between women and men. The other predictors, for example, genetic predisposition (*r* = −0.111, *p* = 0.187), economic affluence (*r* = −0.123, *p* = 0.141), urbanization (*r* = 0.026, *p* = 0.755), and female aging (*r* = 0.091, *p* = 0.277) do not reach statistical significance, suggesting that their independent effects on gender disparity in dementia rates are limited when controlling for the other variables.

Table 3 shows the results from multiple linear regression models (both enter and stepwise) for predicting female dementia incidence and the incidence difference between females and males. In Table 3a, for female dementia incidence, the enter model reveals that the GEI is the strongest predictor (*β* = 0.762, *p* < 0.001), while female aging also has a significant positive effect (*β* = 0.315, *p* < 0.05). In contrast, genetic predisposition, economic affluence, and urbanization do not reach significance. In the stepwise model, GEI remains the only significant predictor (*β* = 0.822, *p* < 0.001) and accounts for 67.4% of the variance (adjusted *R* ^2^ = 0.674).

**TABLE 3:** Multiple linear regression models (enter and stepwise) to identify the significant predictors of dementia incidence rate.

(a) Female dementia incidence							
Dependent variable: Dementia incidence rate, female			Dependent variable: Dementia incidence rate, female				
Predictor	*β*	Sig.	Rank	Predictor	*β*	Sig.	Adjust *R* ^2^
Gender equality index	0.762	< 0.001	1	Gender equality index	0.822	< 0.001	0.674
Genetic predisposition	−0.185	0.092		Genetic predisposition	Not significant		
Economic affluence	−0.122	0.119		Economic affluence	Not significant		
Urbanization, WB 2018	0.037	0.586		Urbanization	Not significant		
Aging, female	0.315	< 0.050		Aging, female	Not significant		

(b) Difference between female and male dementia incidence rate							
Dependent variable: Dementia gender disparity (female–male)			Dependent variable: dementia gender disparity (female–male)				
Predictor	*β*	Sig.	Rank	Predictor	*β*	Sig.	Adjust *R* ^2^
Gender equality index	0.824	< 0.001	1	Gender equality index	0.769	< 0.001	0.589
Genetic predisposition	−0.164	0.187		Genetic predisposition	Not significant		
Economic affluence	−0.131	0.141		Economic affluence	Not significant		
Urbanization	0.024	0.755		Urbanization	Not significant		
Aging, female	0.173	0.277		Aging, female			

*Note:* Data source & definition: Gender Inequality Index (GII), United Nations Development Programme, 2011; Dementia incidence rate (DIR), the number of new cases per 100 000 people diagnosed in 2019 Institute for Health Metrics and Evaluation of University of Washington; Economic affluence, per capita GDP PPP, measured with the per capita purchasing power parity (PPP) value of all final goods and services produced within a territory in a given year (2018), the World Bank; Biological State Index (I_bs_), dementia genetic background accumulation level due to reduced natural selection, downloaded from previous publication (You and Henneberg 2022); Life expectancy at birth, Female (Life e(0)), the World Bank 2018; Urbanization, measured with the percentage of population living in urban area in 2018, the World Bank.

Table 3b examines the difference in dementia incidence between females and males. In the enter model, GEI again emerges as the strongest predictor (*β* = 0.824, *p* < 0.001). The stepwise model confirms this result, with GEI as the sole significant predictor (*β* = 0.769, *p* < 0.001) and an adjusted R^2^ of 0.589. None of the other variables (genetic predisposition, economic affluence, urbanization, or female aging) show significant effects in either model.

Table 4 presents the nonparametric correlations (*ρ*) between the GEI, female dementia incidence, and dementia gender disparity (the difference between female and male incidence) across various country groupings, alongside detailed descriptive statistics for these variables.

**TABLE 4:** Nonparametric correlations and descriptive statistics for gender equality index, female dementia incidence, and dementia gender disparity (female minus male) by country groupings.

	Correlation (nonparametric) between gender equality index, female dementia incidence and dementia gender disparity (female minus male)				Descriptive statistics for independent variable (gender equality index) and dependent variable (female dementia incidence and dementia gender disparity [female minus male])					
Country groupings	Female dementia incidence	*p*	Dementia gender disparity	*p*	Variable	*n*	Minimum	Maximum	Mean	Std. deviation
Worldwide	0.834	< 0.001	0.752	< 0.001	Gender equality index	154	0.21	0.96	0.62	0.19
					Female dementia incidence	154	20.44	595.69	158.39	131.31
					Dementia gender disparity	154	−3.43	296.42	65.86	63.99
World Bank income classifications										
High Income	0.511	< 0.001	0.463	< 0.001	gender equality index	51	0.26	0.96	0.80	0.15
					Female dementia incidence	70	21.66	595.69	256.07	130.60
					Dementia gender disparity	70	−3.43	296.42	108.83	65.44
Upper Middle Income	0.597	< 0.001	0.594	< 0.001	Gender equality index	43	0.40	0.86	0.62	0.12
					Female dementia incidence	55	28.69	328.87	132.82	74.60
					Dementia gender disparity	55	7.38	171.89	53.14	39.03
Low Middle Income	0.864	< 0.001	0.737	< 0.001	Gender equality index	36	0.33	0.72	0.49	0.11
					Female dementia incidence	50	23.25	291.03	74.78	55.67
					Dementia gender disparity	50	−1.95	159.85	30.12	29.55
Low Income	0.389	0.60	0.312	0.138	Gender equality index	24	0.21	0.90	0.43	0.14
					Female dementia incidence	29	18.72	236.57	45.22	40.91
					Dementia gender disparity	29	0.56	108.70	16.47	19.50
Low‐ and middle‐income countries (LMIC)	0.803	< 0.001	0.750	< 0.001	Gender equality index	103	0.21	0.90	0.53	0.14
					Female dementia incidence	134	18.72	328.87	92.21	70.93
					Dementia gender disparity	134	−1.95	171.89	36.62	35.18
Fisher r‐to‐z transformation (LMIC vs. High‐income)	*z* = 3.09, *p* < 0.001		*z* = 2.69, *p* < 0.010							
United Nations (common practice)										
Developed	0.234	0.126	0.118	0.445	Gender equality index	44	0.68	0.96	0.85	0.07
					Female dementia incidence	49	93.90	595.69	321.98	93.17
					Dementia gender disparity	49	29.75	296.42	142.83	52.21
Developing	0.697	< 0.001	0.636	< 0.001	Gender equality index	110	0.21	0.91	0.53	0.14
					Female dementia incidence	155	18.72	361.28	93.57	68.45
					Dementia gender disparity	155	−3.43	160.47	35.65	30.29
Fisher r‐to‐z transformation (Developing vs. developed)	*z* = 3.39, *p* < 0.001		*z* = −3.45, *p* < 0.001							
WHO regions										
Africa	0.616	< 0.001	0.580	< 0.001	Gender equality index	36	0.33	0.90	0.43	0.11
					Female dementia incidence	47	18.72	189.61	45.99	31.41
					Dementia gender disparity	47	3.08	84.30	20.74	16.50
Americas	0.598	< 0.001	0.631	< 0.001	Gender equality index	29	29	0.38	0.87	0.59
					Female dementia incidence	29	38	39.85	337.60	148.85
					Dementia gender disparity	29	38	7.21	135.56	54.28
Eastern Mediterranean	0.356	0.135	0.095	0.700	Gender equality index	19	0.21	0.82	0.51	0.19
					Female dementia incidence	22	21.66	196.02	66.77	44.69
					Dementia gender disparity	22	−3.43	61.19	15.96	16.86
Europe	0.453	< 0.001	0.347	< 0.050	Gender equality index	49	0.58	0.96	0.82	0.10
					Female dementia incidence	54	74.17	535.60	290.47	104.92
					Dementia gender disparity	54	31.03	245.63	128.36	54.90
South‐East Asia	0.214	0.610	0.467	0.233	Gender equality index	8	0.41	0.65	0.53	0.08
					Female dementia incidence	11	47.03	207.25	97.57	54.08
					Dementia gender disparity	11	2.61	114.32	38.18	34.05
Western Pacific	0.896	< 0.001	0.786	< 0.001	Gender equality index	13	0.33	0.91	0.67	0.18
					Female dementia incidence	32	30.46	595.69	132.33	112.97
					Dementia gender disparity	32	6.59	296.42	55.78	55.60
Countries grouped with various factors										
Asia Cooperation Dialogue	0.362	0.064	0.432	< 0.050	Gender equality index	27	0.26	0.91	0.61	0.17
					Female dementia incidence	30	21.66	595.69	120.17	116.54
					Dementia gender disparity	30	−3.43	296.42	50.04	61.44
Asia‐Pacific Economic Cooperation	0.822	< 0.001	0.744	< 0.001	Gender equality index	17	0.33	0.91	0.70	0.16
					Female dementia incidence	20	36.88	595.69	192.13	135.20
					Dementia gender disparity	20	6.59	296.42	81.29	67.39
Arab World	0.350	0.155	0.117	0.645	Gender equality index	18	0.21	0.82	0.52	0.18
					Female dementia incidence	21	21.66	196.02	67.88	44.07
					Dementia gender disparity	21	−3.43	61.19	16.58	17.28
European Economic Area	0.044	0.819	0.088	0.648	Gender equality index	29	0.68	0.96	0.86	0.07
					Female dementia incidence	29	208.26	535.60	346.42	78.22
					Dementia gender disparity	29	69.07	245.63	156.08	46.49
European Union	0.163	0.418	0.030	0.881	Gender equality index	27	0.68	0.96	0.86	0.07
					Female dementia incidence	27	208.26	535.60	350.79	79.26
					Dementia gender disparity	27	69.07	245.63	160.01	45.52
English as Official Language	0.810	< 0.001	0.776	< 0.001	Gender equality index	39	0.33	0.95	0.59	0.20
					Female dementia incidence	57	23.25	371.54	115.02	92.26
					Dementia gender disparity	57	0.82	165.92	45.47	38.27
Latin America	0.506	< 0.050	0.499	< 0.050	Gender equality index	22	0.38	0.69	0.56	0.07
					Female dementia incidence	24	39.85	321.90	130.93	68.23
					Dementia gender disparity	24	7.21	135.56	46.56	31.79
Latin America and Caribbean	0.502	< 0.010	0.548	< 0.010	Gender equality index	27	0.38	0.69	0.58	0.07
					Female dementia incidence	35	39.85	321.90	134.84	62.00
					Dementia gender disparity	35	7.21	135.56	48.86	28.14
Organization for Economic Co‐operation & Development	0.383	< 0.050	0.285	< 0.092	Gender Equality Index	36	0.55	0.96	0.85	0.10
					Female Dementia Incidence	37	99.70	595.69	324.25	104.72
					Dementia Gender Disparity	37	29.21	296.42	143.30	59.09
Southern African Development Community	0.533	0.061	0.467	0.108	Gender Equality Index	13	0.34	0.65	0.45	0.08
					Female Dementia Incidence	16	29.11	189.61	62.99	44.20
					Dementia Gender Disparity	16	11.15	84.30	32.21	20.93
Shanghai Cooperation Organization	0.462	< 0.050	0.454	< 0.050	Gender Equality Index	25	0.26	0.86	0.59	0.17
					Female Dementia Incidence	26	21.66	285.02	107.66	79.61
					Dementia Gender Disparity	26	−3.43	171.89	44.55	46.62

*Note:* Data source & definition: Gender Inequality Index (GII), United Nations Development Programme, 2011; Dementia incidence rate (DIR), the number of new cases per 100 000 people diagnosed in 2021 Institute for Health Metrics and Evaluation of University of Washington.

Worldwide, GEI is strongly correlated with FDIR (*ρ* = 0.834, *p* < 0.001) and with gender disparity (*ρ* = 0.752, *p* < 0.001), indicating that GEI is linked to increased female dementia rates and a wider gap between female and male incidence. Descriptively, on a global scale, the GEI values range from 0.21 to 0.96, with a mean of 0.62 and a standard deviation (SD) of 0.19. This moderate average, along with the wide range, highlights considerable variation in gender equality across countries. Female dementia incidence ranges from 20.44 to 595.69 new cases per 100 000, with a mean of 158.39 and a large SD of 131.31, indicating substantial variability in dementia rates among women. Similarly, dementia gender disparity ranges from −3.43 to 296.42, with a mean of 65.86 (SD = 63.99), suggesting that on average, women experience significantly higher dementia rates than men, although some countries show a reversed or negligible disparity.

When countries are grouped by World Bank income classifications, the associations remain strong in LMICs, where GEI correlates with FDIR at *ρ* = 0.803 and with gender disparity at *ρ* = 0.750 (both *p* < 0.001). Fisher *r*‐to‐*z* transformations indicate significant differences between LMICs and high‐income countries (*z* = 3.09, *p* < 0.001 for FDIR; *z* = −2.69, *p* < 0.010 for gender disparity). Descriptive statistics further underscore these differences; for example, high‐income countries report a higher mean GEI (approximately 0.80) and higher female dementia incidence (mean around 256.07), while LMICs have a lower mean GEI (about 0.53) and a lower mean FDIR (approximately 92.21).

Grouping countries by United Nations classification reveals that developed nations exhibit weaker and often non‐significant correlations (FDIR: *ρ* = 0.234, *p* = 0.126; gender disparity: *ρ* = 0.118, *p* = 0.445) along with higher mean GEI values (mean = 0.85, SD = 0.07), and elevated FDIR (mean = 321.98, SD = 93.17) and gender disparity (mean = 142.83, SD = 52.21). In contrast, developing countries show strong associations (FDIR: *ρ* = 0.697, *p* < 0.001; gender disparity: *ρ* = 0.636, *p* < 0.001) with lower mean GEI (mean = 0.53, SD = 0.14), FDIR (mean = 93.57, SD = 68.45), and gender disparity (mean = 35.65, SD = 30.29).

Regional analyses across WHO regions also reveal varying strengths of correlations and differences in descriptive statistics, likely due to differences in sample sizes and country homogeneity. Overall, these findings underscore the substantial role of gender equality in shaping female dementia incidence and gender disparities, while the detailed descriptive statistics highlight significant variability in these measures across different economic and regional contexts.

## 4 Discussion

This study reveals a paradoxical link between gender equality and women's dementia risk. Analysis of 154 countries showed that higher GEI scores were significantly associated with both increased female dementia incidence and greater gender disparity. While gender equality enhances social and economic empowerment, it may also expose women to new dementia‐related risks through lifestyle changes, occupational stress, and psychosocial pressures.

### 4.1 Gender Equality, Lifestyle, and Stress

In high‐GEI countries, women's expanded access to education and employment has contributed to significant lifestyle shifts. While beneficial in many respects, these shifts include increased participation in high‐stress occupations, which may elevate chronic stress exposure, a well‐documented risk factor for cognitive decline and dementia (Antipas et al. 2024; Lupien et al. 2009; Yaffe et al. 2010). Additionally, women in these settings are more likely to adopt behaviors traditionally associated with men, such as higher alcohol consumption and smoking (Gupta and Warner 2008; Muhammad et al. 2021; Park et al. 2013; Verplaetse et al. 2025). Both are strongly linked to vascular dysfunction and neurotoxicity, exacerbating dementia risk through mechanisms such as cerebrovascular damage and neuronal loss (Lu et al. 2021; Shang et al. 2022; Wang et al. 2024). By contrast, women in low‐GEI countries often maintain physically active lifestyles through agricultural, domestic, or manual labor roles, which confer cardiovascular and cognitive protection (Liu et al. 2022). Traditional diets in these regions, typically less processed and more plant‐based, further support metabolic and vascular health, reducing dementia‐related risk factors (Ding et al. 2022; Iso‐Markku et al. 2022).

### 4.2 Cultural Norms, Relationships, and Caregiving

Cultural practices also shape dementia risk. In low‐GEI settings, prevailing norms around lifelong monogamy and religiously reinforced family cohesion may reduce exposure to relationship transitions that are associated with psychological stress and depression, both risk factors for dementia (Girme et al. 2023; Wallensten et al. 2023). In contrast, high‐GEI countries, where evolving gender roles have increased acceptance of sequential or multiple partnerships, women are disproportionately affected by relationship‐related emotional distress and caregiving burdens (Apostolou et al. 2023; Aviv et al. 2025; Delgado‐Herrera et al. 2024). Even in equitable societies, women continue to assume the dual responsibilities of caregiving and professional work (Stall et al. 2023). This “double load” sustains chronic stress and accelerates pathways of hippocampal atrophy linked to dementia (Kwon 2020; Lupien et al. 2009).

### 4.3 Biological and Demographic Influences

Although our analysis controlled for female life expectancy, residual biological influences likely persist. Women's longer lifespans naturally increase their dementia risk (Levine et al. 2021), and estrogen decline after menopause remains an important contributor (Ali et al. 2023). In high‐GEI contexts, smaller family sizes and delayed childbirth reduce cumulative exposure to endogenous estrogen, further heightening dementia susceptibility (Gong et al. 2022). Demographic transitions common in affluent countries, such as declining fertility rates, may also influence genetic diversity and natural selection pressures, potentially increasing population‐level vulnerability to dementia (Budnik and Henneberg 2017; You and Henneberg 2016, 2017; You et al. 2018).

### 4.4 Structural Determinants of Dementia

Our findings underscore the role of gender equality as a structural determinant of dementia risk (UNDP 2024; Yu et al. 2023). While improved GEI typically coexists with urbanization and affluence, these transitions are often accompanied by environmental and behavioral risks such as sedentary lifestyles, processed diets, and pollution exposure (Huque et al. 2023; Kornblith et al. 2022). In this context, the benefits of enhanced healthcare access and diagnostic accuracy may be offset by increased prevalence of risk‐enhancing exposures. Additionally, ethnically diverse populations in high‐GEI countries may contribute to the observed patterns. For example, African American and Hispanic women in the U.S. demonstrate higher dementia incidence than non‐Hispanic White women, largely due to cardiovascular risk factors, diabetes, and socioeconomic disparities (Lennon et al. 2022; Vemuri et al. 2014). These group‐specific vulnerabilities amplify the aggregate dementia risk among women in high‐GEI societies.

### 4.5 Underdiagnosis in Low‐GEI Countries

A critical caveat is the likelihood of underdiagnosis in low‐GEI settings. Cognitive decline is often dismissed as normal aging, and women's dementia symptoms are frequently overlooked or attributed to natural frailty (Gamble et al. 2022; Guruprasad 2024; Ribeiro et al. 2023; Sadhwani 2023; Thomas et al. 2023). Limited healthcare infrastructure and cultural stigmas surrounding cognitive impairment further suppress detection rates. Consequently, the true dementia incidence in low‐GEI countries may be substantially higher than reported, which could partly explain the observed association between GEI and female dementia burden.

### 4.6 Confounding Factors and Interpretative Caution

Although GEI emerged as the most significant predictor of FDIR and gender disparity, other factors such as economic affluence, urbanization, female aging, and genetic predisposition may exert modifying influences. For instance, affluence correlates with improved healthcare access and diagnostic accuracy (Huque et al. 2023; You et al. 2018), potentially inflating reported dementia incidence in high‐GEI countries. Urbanization, also closely tied to GEI, introduces risk factors such as reduced physical activity and greater exposure to unhealthy diets and pollutants (Kornblith et al. 2022). Similarly, aging and genetic predisposition interact with social and environmental exposures, influencing dementia risk trajectories. While statistically controlled in our models, these variables remain interdependent, and residual confounding cannot be excluded.

## 5 Conclusions

This study provides novel evidence that rising gender equality, while socially beneficial, may paradoxically increase female dementia incidence and gender disparity. By highlighting the intersection of lifestyle, cultural norms, biological factors, and structural determinants, our findings advance understanding of global dementia risk patterns and underscore the need for gender‐informed, context‐sensitive public health strategies. Future research employing longitudinal or individual‐level data will be critical to disentangle causal pathways and inform effective interventions.

### 5.1 Public Health and Policy Implications

The paradoxical link between gender equality and dementia underscores the need for gender‐sensitive prevention strategies. In high‐GEI settings, interventions should focus on mitigating stress, reducing modifiable risk behaviors (e.g., alcohol and tobacco use), and supporting women balancing caregiving with professional roles. In low‐GEI countries, improved diagnostic infrastructure, culturally sensitive health education, and community‐based awareness campaigns are essential to reduce underdiagnosis. Policymakers must recognize that structural advances in gender equality may inadvertently heighten dementia risk through lifestyle and demographic shifts. Preventive strategies must therefore integrate both equity goals and cognitive health promotion to ensure that social progress does not unintentionally contribute to health inequities.

### 5.2 Strengths and Limitations

This study utilizes global data from the World Bank, IHME, and previous publications to explore the relationship between the GEI, female dementia incidence rates (FDIR), and gender disparity in dementia incidence. A key strength of this study is its ecological design, which enables a broad, population‐level analysis of gender equality's impact on dementia patterns worldwide. Conducting a study of this scale at the individual level would be nearly impossible due to the relatively low incidence of dementia (IHME 2020), which requires vast sample sizes to capture meaningful trends across diverse countries and demographics. Additionally, measuring the GEI at an individual level is challenging, as it represents complex societal factors that cannot be easily quantified on a personal scale. Thus, this approach provides insights that would not be feasible through individual‐level studies.

However, this study has several limitations. The ecological design, while useful for identifying population‐level associations, poses the risk of ecological fallacy, where observed trends may not directly apply to individuals. Additionally, the cross‐sectional nature of the data limits the ability to establish causal relationships between GEI and dementia incidence, making it difficult to determine whether gender equality directly influences dementia risk over time. We acknowledge that while our findings reveal strong associations, they do not determine the temporal sequence of these relationships or confirm causation. Future research should employ longitudinal and cohort study designs to track changes in gender equality, lifestyle behaviors, and dementia incidence over time. Additionally, integrating mixed‐method approaches and individual‐level data could provide deeper insights into the mechanisms linking gender equality to dementia risk. These approaches will enhance our understanding of how evolving gender roles impact women's cognitive health.

The reliance on aggregated data also presents challenges. In low‐GEI countries, limited healthcare infrastructure, lower levels of healthcare access, lower awareness of dementia, and reliance on informal caregiving contribute to significant underdiagnosis and underreporting, particularly among women (Seeher et al. 2023). Cultural norms often normalize cognitive decline as a natural part of aging, leading to delayed recognition and diagnosis. This underreporting likely results in underestimated female dementia incidence, potentially affecting the observed association between GEI and dementia risk. We have clarified this issue and included references to support this discussion.

Another limitation is the inability to control for individual‐level risk factors such as smoking, alcohol use, physical inactivity, and diet, which are well‐established contributors to dementia risk. Since this study employs an ecological approach using population‐level data, controlling for these variables was not feasible. However, we emphasize the need for future research incorporating individual‐level datasets to assess these personal risk factors more precisely. We recommend longitudinal and cohort studies that integrate both macro‐ and micro‐level influences to refine our understanding of how gender equality impacts dementia risk.

While global datasets offer broad coverage, they lack the granularity needed to capture regional variations and socio‐cultural influences on dementia patterns. Region‐specific analyses and mixed‐method approaches will further clarify how gender equality interacts with diverse socio‐economic and cultural factors to shape cognitive health outcomes.

## Author Contributions

**Wenpeng You:** conceptualization, methodology, software, data curation, investigation, validation, formal analysis, funding acquisition, visualization, project administration, resources, writing – review and editing, writing – original draft.

## Ethics Statement

The data used in this study are secondary and freely accessible from previous publications and repositories of the World Health Organization, the World Bank, and Health Metrics and Evaluation (IHME). As the data are only identifiable to the population level and not to individuals or communities, ethical approval or written informed consent was not necessary. This study was exempted from ethical approval by the Office of Research Ethics, Compliance, and Integrity (ORECI) at the University of Adelaide (Ethics Approval Number: 36289).

## Conflicts of Interest

Wenpeng You is an Associate Editor for Nursing and Health Sciences and a lead author on this article. The manuscript was managed by editors unaffiliated with the author or institution and monitored carefully to ensure there is no peer review bias.

## Supporting information


**Data S1:** Data selection flowchart for analysis.

## Data Availability

The data that support the findings of this study are available from the corresponding author upon reasonable request.
