# Supplementary material for: Dementia Risk and Gender Equality: Global Insights Into Social Determinants
Source: Nurs Health Sci. 2025 Sep 16;27(3):e70230. doi: 10.1111/nhs.70230 (PMC12441479; doi:10.1111/nhs.70230)
Supplement: Supplementary file 1 — Data S1: Data selection flowchart for analysis. [file NHS-27-e70230-s001.docx]

**Supplemental File 1: Data Selection Flowchart for Analysis**

**1. Data Identification**

- **Gender Equality Index (GEI):** Derived from the United Nations Development Programme (2011).
- **Dementia Incidence Rates:** Extracted from the Institute for Health Metrics and Evaluation (IHME, 2021).
- **Genetic Predisposition:** Sourced from prior published data (2022).
- **Economic Affluence:** Based on GDP per capita (World Bank, 2018).
- **Urbanization Levels:** Percentage of urban population (World Bank, 2018).
- **Female Aging:** Life expectancy at birth (World Bank, 2018).

**2. Country Selection Process**

- **Step 1:** Extracted **204 countries/territories** with available dementia incidence data (IHME, 2021).
- **Step 2:** Matched with **GEI data** (United Nations Development Programme, 2011).
- **Step 3:** **Applied Inclusion Criteria** – Countries were included if they had:
  - Female and male dementia incidence data.
  - Corresponding GEI and confounding variables (genetic predisposition, economic indicators).
- **Step 4:** **Applied Exclusion Criteria** – Countries missing key variables were excluded to ensure consistency.
- **Step 5:** Final dataset included **148–198 countries**, depending on variable availability.

**3. Variable Integration & Data Processing**

- **Dependent Variables:**
  - Female Dementia Incidence Rate (FDIR).
  - Dementia Gender Disparity (Female - Male).
- **Independent Variable:**
  - Gender Equality Index (GEI).
- **Confounding Variables:**
  - Genetic Predisposition, Economic Affluence, Urbanization, Female Aging.
- **Data Processing:**
  - Matched variables across selected countries.
  - Ensured consistency in measurement across datasets.

**4. Statistical Analysis**

- **Correlation Analysis:** Examined relationships among variables.
- **Regression Analysis:** Assessed GEI as a predictor of dementia incidence and gender disparity.
- **Subgroup Analysis:** Compared trends across income levels, regions, and socio-economic classifications.
